# Supplementary material for: Stress Exposure of Evolved Bacteriophages under Laboratory versus Food Processing Conditions Highlights Challenges in Translatability
Source: Viruses. 2022 Dec 30;15(1):113. doi: 10.3390/v15010113 (PMC9865000; doi:10.3390/v15010113)
Supplement: Supplementary file 1 [file viruses-15-00113-s001.zip › viruses-2020638-supplementary.pdf]

Stress Exposure of Evolved Bacteriophages under Laboratory versus Food Processing Conditions Highlights Challenges in Translatability

Mellissa Gomez [1], Alexandra Szewczyk [1], Jake Szamosi [2], Vincent Leung [1], Carlos Filipe [1,*] and Zeinab Hosseinidoust [1,3,4,*]

[1] Department of Chemical Engineering, McMaster University, Hamilton, ON L8S 4L7, Canada
[2] Department of Medicine, McMaster University, Hamilton, ON L8P 1H6, Canada
[3] School of Biomedical Engineering, McMaster University, Hamilton, ON L8S 4K1, Canada
[4] Michael DeGroote Institute for Infectious Disease Research, McMaster University, Hamilton, ON L8S 4K1, Canada
* Correspondence: filipec@mcmaster.ca (C.F.); doust@mcmaster.ca (Z.H.)

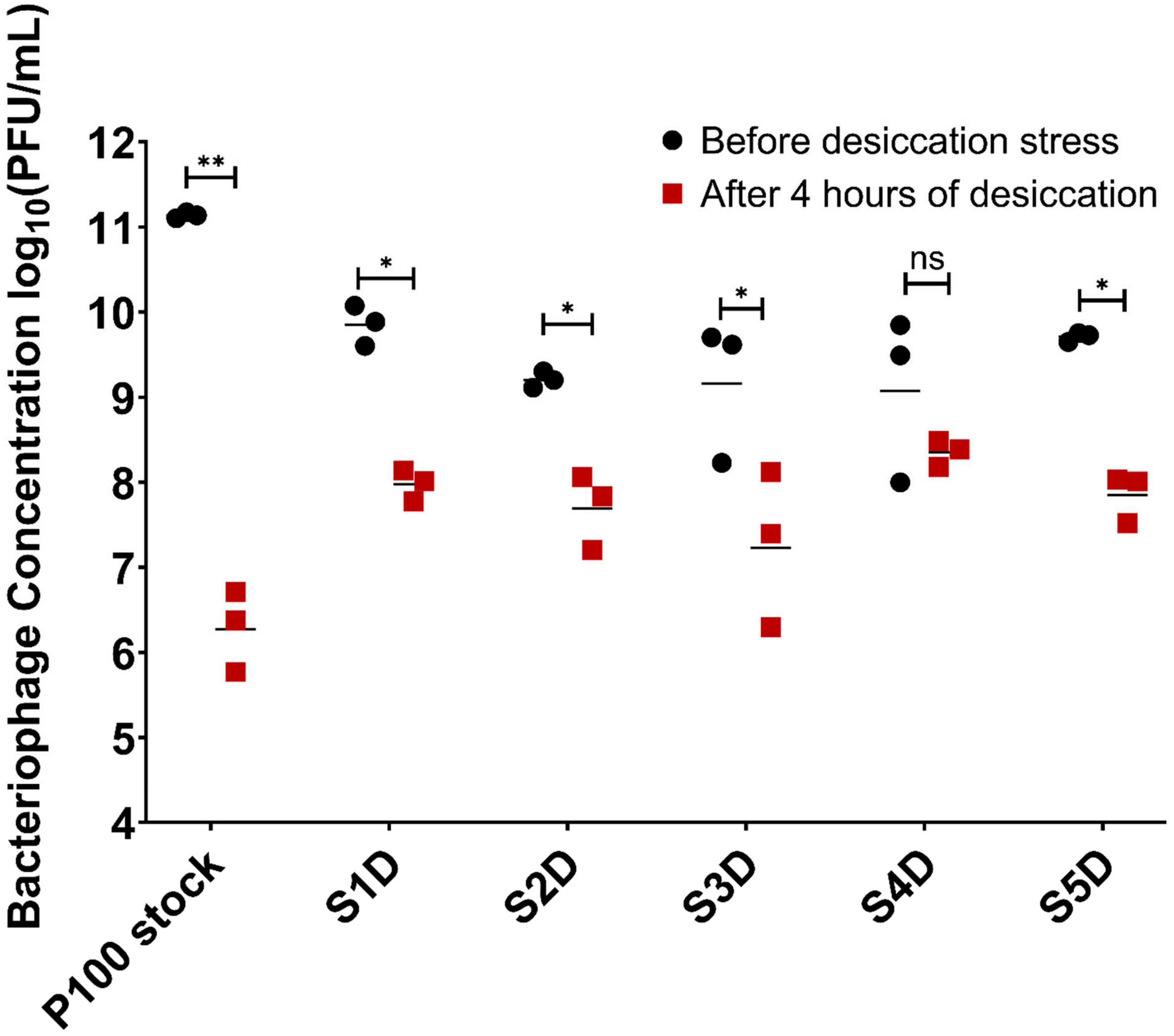

Figure S1. Measured concentrations of viable bacteriophage in the P100 stock as compared to samples that have undergone 1-5 cycles of selection before and after exposure to desiccation for 4 hrs. Results are shown as the individual results of triplicate experiment, where the geometric mean has been marked. Bacteriophage concentration prior to exposure presented in black and concentration after exposure presented in red. Significant differences in bacteriophage concentration before and after exposure to desiccation are marked as * - $p \leq 0.05$ and ** - $p \leq 0.01$. Nomenclature: n.s.: not significant; S1D: P100 sample that underwent 1 cycle of desiccation selection; S2D: P100 sample that underwent 2 cycles of desiccation selection; S3D: P100 sample that underwent 3 cycles of desiccation selection; S4D: P100 sample that underwent 4 cycles of desiccation selection S5D: P100 sample that underwent 5 cycles of desiccation selection.

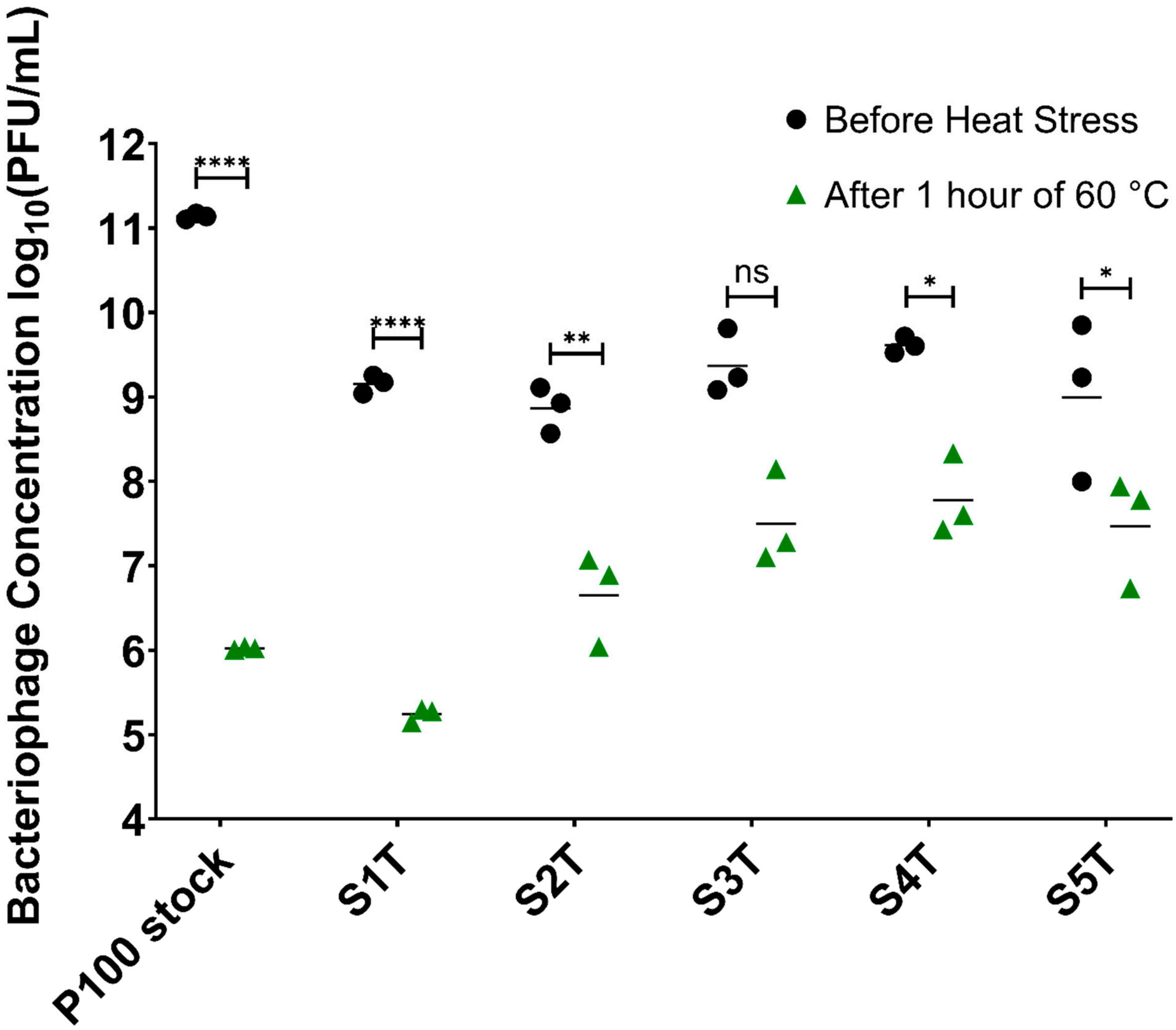

Figure S2. Measured concentrations of viable bacteriophage in the P100 stock as compared to samples that have undergone 1-5 cycles of selection before and after exposure to temperatures of 60 °C of 1 hr. Results are shown as the individual results of triplicate experiment, where the geometric mean has been marked. Bacteriophage concentration prior to exposure presented in black and concentration after exposure presented in green. Significant differences in bacteriophage concentration before and after exposure to 60 °C are marked as * - $p \leq 0.05$, ** - $p \leq 0.01$, and **** - $p \leq 0.0001$. Nomenclature: n.s.: not significant; S1T: P100 sample that underwent 1 cycle of elevated temperature selection; S2T: P100 sample that underwent 2 cycles of elevated temperature selection; S3T: P100 sample that underwent 3 cycles of elevated temperature selection; S4T: P100 sample that underwent 4 cycles of elevated temperature selection S5T: P100 sample that underwent 5 cycles of elevated temperature selection.

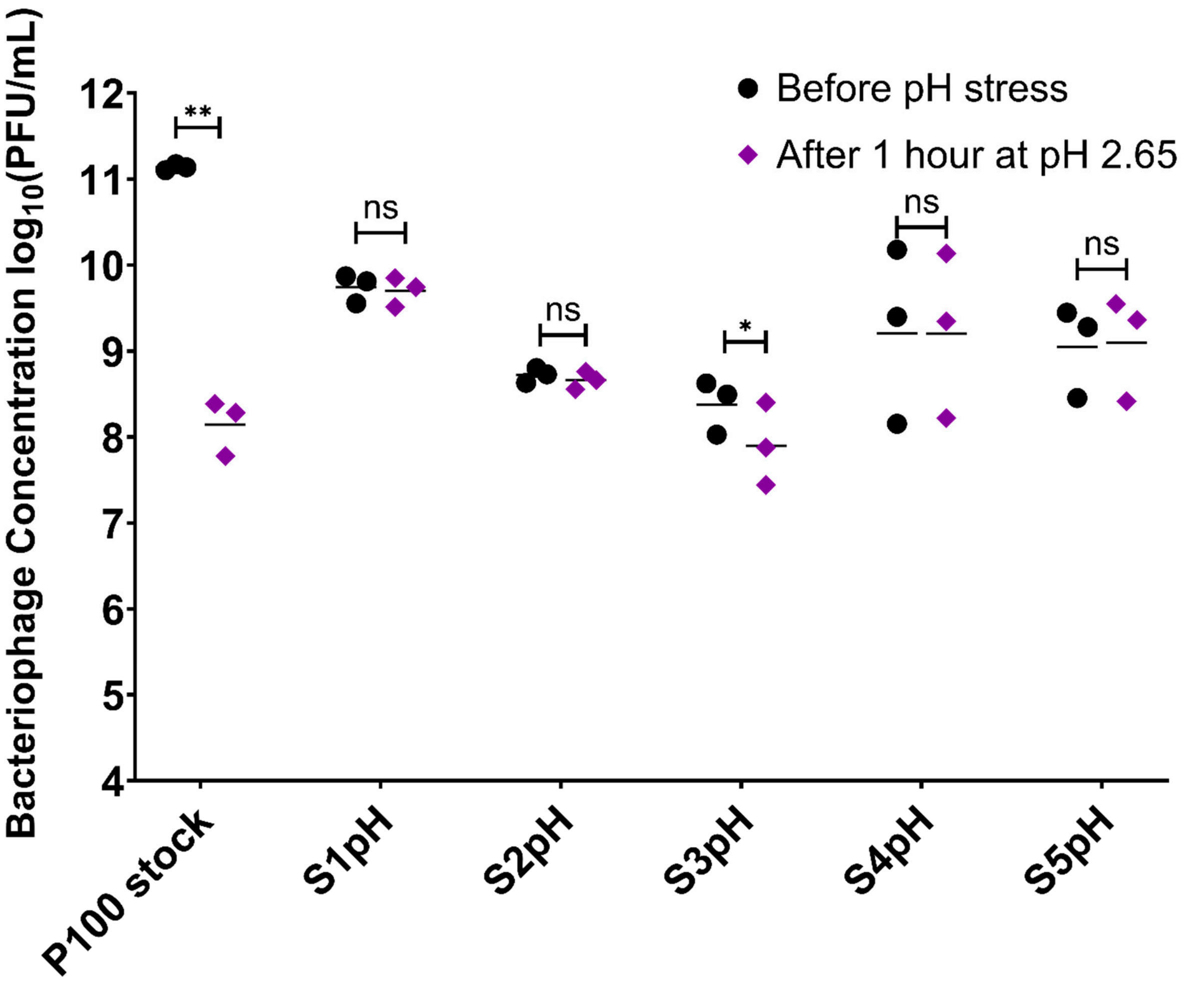

Figure S3. Measured concentrations of viable bacteriophage in the P100 stock as compared to samples that have undergone 1-5 cycles of selection before and after exposure to pH of 2.65 for 1 hr. Results are shown as the individual results of triplicate experiment, where the geometric mean has been marked. Bacteriophage concentration prior to exposure presented in black and concentration after exposure presented in purple. Significant differences in bacteriophage concentration before and after exposure to 60 °C are marked as * - $p \leq 0.05$ and ** - $p \leq 0.01$. Nomenclature: n.s.: not significant; S1D: P100 sample that underwent 1 cycle of low-pH selection; S2D: P100 sample that underwent 2 cycles of low-pH selection; S3D: P100 sample that underwent 3 cycles of low-pH selection; S4D: P100 sample that underwent 4 cycles of low-pH selection S5D: P100 sample that underwent 5 cycles of low-pH selection.

Table S1 Analogues to key P100 genomic regions affected by selection found in other listeria bacteriophages. Protein functions are given if previously determined. Abbreviations: GP: gene product; HP: hypothetical protein, NCR: non-coding region

Bacteriophage	P100 regions					Reference
	GP39	GP40	GP102	GP108	NCR Position 95,836-95,947	
20422-1				HP		(1)
List-36	HP	HP	HP	HP		Rajanna, C., et al. (2014) Direct Submission
LMSP-25	HP	HP	HP	HP		Woolston, J., et al. (2014) Direct Submission
LMTA-148	HP	HP	HP		NCR	Woolston, J., et al. (2014) Direct Submission
LMTA-34	HP	HP	HP	HP	NCR	Woolston, J., et al. (2014) Direct Submission
LMTA-57		HP	HP	HP	NCR	Woolston, J., et al. (2014) Direct Submission
LMTA-94	HP			HP	NCR	Woolston, J., et al. (2014) Direct Submission
LP-039					NCR	(2)
LP-048	HP	HP	HP		NCR	(3)
LP-064		HP			NCR	(3)
LP-066					NCR	(2)
LP-083-2	HP	HP			NCR	(3)
LP-124					NCR	(3)
LP-125	HP	HP	HP	HP	NCR	(3)
LP-Mix_6.1		HP			NCR	(4)
LP-Mix_6.2	HP	HP			NCR	(4)
vB_Lino_VEfB7	Receptor binding protein	Tail fiber assembly chaperone	HP	AntiCRISPR endonuclease		(5)
vB_Liva_VAfA18	Tail fiber protein	Tail fiber assembly chaperone	HP	AntiCRISPR endonuclease		(5)
vB_LmoM_AG20	HP	HP	HP		NCR	Anany, H., et al. (2012) Direct Submission
A511	Receptor binding protein	Assembly chaperone	HP	HP	NCR	(6)
WIL-1					NCR	Silva-Castro, G.A., et al. (2014) Direct Submission
P100plus	Receptor binding protein	HP		HP	NCR	Dunne, M., et al. (2020), Direct Submission

P200	Receptor binding protein	Assembly chaperone			NCR	Dunne, M., et al. (2020), Direct Submission
P61	HP	HP	HP	HP	NCR	(7)

Table S2 Efficiency of plaquing for the different P100 bacteriophage samples infecting the *L. monocytogenes* serotype 1/2a and 4b samples. *L. monocytogenes* serotype 1/2a was used as the reference bacterial strain. Statistical analysis showed no significant difference in the infectivity of the *L. monocytogenes* 4b sample with the conditioned bacteriophage strains as compared to the ancestral P100 stock. Three biological replicates were tested for the different engineered P100 samples, with all experiments being performed in triplicate. Results are shown as average of the biological replicates ± standard deviation for the conditioned phage and the average of the technical replicates ± standard deviation for the P100 stock. Nomenclature: R5D: P100 sample that underwent 5 cycles of desiccation selection followed by 5 propagation cycles in the absence of stress; R5T: P100 sample that underwent 5 cycles of high temperature selection followed by 5 propagation cycles in the absence of stress, R5pH: P100 sample that underwent 5 cycles of low pH selection followed by 5 propagation cycles in the absence of stress**.**

**Bacteriophage Sample**	***L. monocytogenes* 1/2a**	***L. monocytogenes* 4b**
P100 stock	1	0.51 ± 0.21
R5D	1	0.70 ± 0.22
R5T	1	0.72 ± 0.09
R5pH	1	0.49 ± 0.06

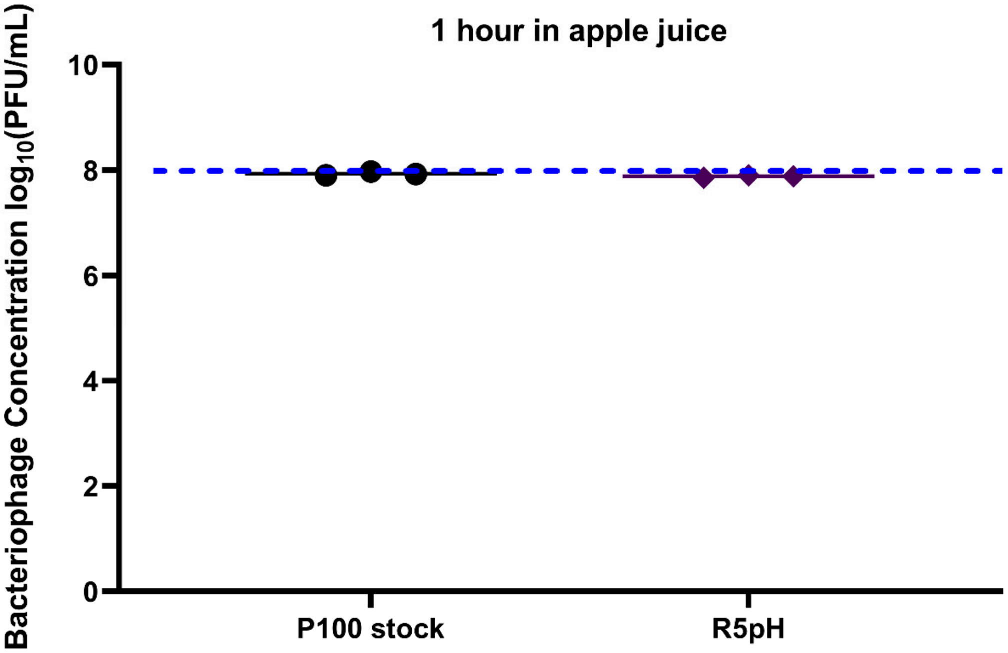

Figure S4. Remaining bacteriophage concentration (PFU/ml), of P100 stock and the reversion-tested phage samples R5pH after exposure to apple juice for 1 hour. Triplicate results are shown, where the geometric mean is marked as a horizontal line. Black circles represent the P100 ancestral stock and purple diamonds represent the R5pH sample. The horizontal dotted blue line represents the initial concentration of $1x10^8$ PFU/ml for the P100 and R5pH samples. Nomenclature: R5pH: S5pH sample that was propagated 5 times in the absence of stress.

References

1. Kim JW, Siletzky RM, Kathariou S. Host ranges of Listeria-specific bacteriophages from the Turkey processing plant environment in the United States. Appl Environ Microbiol. 2008;74(21):6623–30.

2. Peters TL, Hudson LK, Song Y, Denes TG. Complete Genome Sequences of Two Listeria Phages of the Genus Pecentumvirus. Dennehy JJ, editor. Microbiol Resour Announc [Internet]. 2019 Nov 14;8(46):1229–48. Available from: http://mra.asm.org/

3. Denes T, Vongkamjan K, Ackermann H-W, Switt AIM, Wiedmann M, Bakker HC den. Comparative Genomic and Morphological Analyses of Listeria Phages Isolated from Farm Environments. Appl Environ Microbiol [Internet]. 2014 [cited 2021 Jul 20];80(15):4616. Available from: /pmc/articles/PMC4148797/

4. Peters TL, Song Y, Bryan DW, Hudson LK, Denes TG. Mutant and Recombinant Phages Selected from In Vitro Coevolution Conditions Overcome Phage-Resistant Listeria monocytogenes. Dudley EG, editor. Appl Environ Microbiol [Internet]. 2020 Oct 28;86(22). Available from: https://doi.org/10.1128/AEM

5. Blanco Fernandez MD, Klumpp J, Barrios ME, Mbayed VA. Gene gain and loss and recombination shape evolution of Listeria bacteriophages of the genus Pecentumvirus. Genomics

[Internet]. 2021;113(1):411–9. Available from: https://doi.org/10.1016/j.ygeno.2020.12.003

6. Klumpp J, Dorscht J, Lurz R, Bielmann R, Wieland M, Zimmer M, et al. The terminally redundant, nonpermuted genome of Listeria bacteriophage A511: A model for the SPO1-like myoviruses of gram-positive bacteria. J Bacteriol [Internet]. 2008 Sep [cited 2021 Jul 21];190(17):5753–65. Available from: http://jb.asm.org/.

7. Stone E, Lhomet A, Neve H, Grant IR, Campbell K, McAuliffe O. Isolation and Characterization of Listeria monocytogenes Phage vB_LmoH_P61, a Phage With Biocontrol Potential on Different Food Matrices. Front Sustain Food Syst. 2020 Nov 5;0:205.
